# Magnetic Resonance Imaging in Case of Cortical Apex Syndrome Caused by Varicella Zoster Virus

**DOI:** 10.2174/1874364100802010109

**Published:** 2008-06-03

**Authors:** Suguru Shirato, Toshiyuki Oshitari, Katsuhiro Hanawa, Emiko Adachi-Usami

**Affiliations:** 1 Department of Ophthalmology, Center for Sensory Organ Diseases, Sannoh Medical Center, Chiba, Japan; 2 Department of Ophthalmology, Kimitsu Central Hospital, Kisarazu, Japan

**Keywords:** Orbital apex syndrome, varicella zoster virus, magnetic resonance imaging, visually evoked cortical potentials.

## Abstract

We present a case of orbital apex syndrome that developed after a re-activation of varicella zoster virus. The magnetic resonance imaging showed signs of a diffuse inflammation of the ipsilateral orbital cavity, external ocular muscles and optic nerve. Pattern visually evoked cortical potentials were non-recordable by stimulating the affected eye.

## INTRODUCTION

Reactivated varicella zoster virus (VZV) can cause herpes zoster ophthalmicus (HZO) in approximately 50% of the cases [1]. However, the development of ophthalmoplegia [2-5] and optic neuritis [6-9] is relatively rare in cases of HZO. During the acute eruptive phase of HZO, it is often difficult to detect abnormalities of the eye because of a complete levator paralysis with severe dermatitis. Thus, an accurate diagnosis may not be possible.

We report a case of orbital apex syndrome that developed 6 days after the onset of HZO. The orbital apex syndrome is defined as an optic neuropathy associated with complete ophthalmoplegia and paralysis in the distribution of the branches of the ophthalmic nerve. We showed that magnetic resonance imaging (MRI) in our case of orbital apex syndrome showed signs of a diffuse inflammation of the ipsilateral orbital cavity, ocular muscles, and optic nerve. These changes have not been previously reported in cases of orbital apex syndrome.

## CASE REPORT

A 71-year-old man, who had colon carcinoma, visited his internist because of headaches and pain around the right eye. Roxoprofen, an anti-inflammatory agent, was prescribed. After 6 days, the patient was referred to our clinic because he was suspected to have early herpes zoster ophthalmicus (HZO). After informing him of the examination procedures, an informed consent was obtained to perform the tests.

External examination showed severely erupted skin lesions over the right ophthalmic branch (V_1_) of the trigeminal nerve. His visual acuity could not be measured because of a complete levator paralysis with severe dermatitis. However, when the eyelids were held open with an instrument (Desmar), a mild pseudo-dendritic keratitis was detected. Acyclovir eye ointment and antibiotic eyedrops were prescribed.

After 12 days, the eruptions on the skin and the swelling of the upper eyelid were slightly improved. But then a total ophthalmoplegia developed in the right eye. The pupil was dilated to 7 mm and did not react to light and accommodation. The cornea was clear although cells were seen in the anterior chamber. The fundus and optic disc were normal, however his visual acuity was reduced to 0.1.

**Fig. (1):**
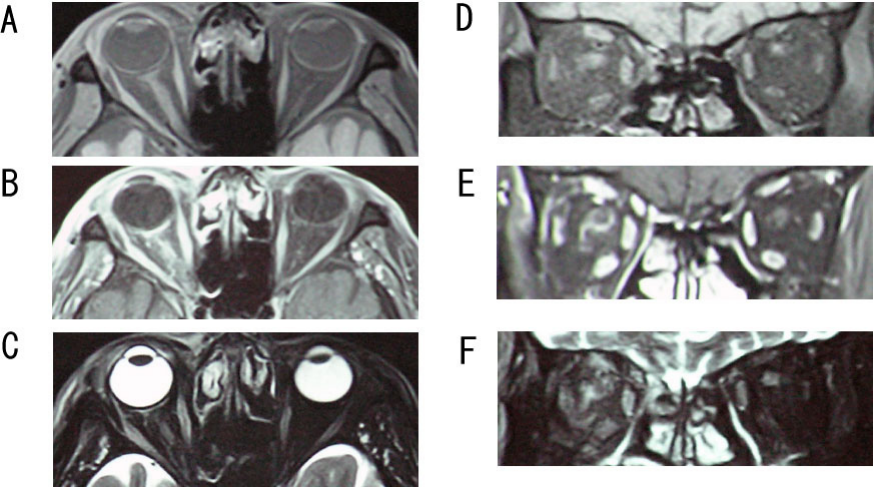
Images obtained by magnetic resonance imaging (MRI) in an eye with orbital apex syndrome. **(A)** Horizontal MRI, T_1_-weighted fat suppressed image. **(B)** Horizontal MRI, T_1_-weighted with Gadolinium enhanced image. **(C)** Horizontal MRI, T_2_-weighted and horizontal image. **(D)** Coronal MRI, T_1_-weighted fat suppressed image. **(E)**. Coronal MRI, T_1_-weighted with Gadolinium enhanced image. **(F)** Coronal MRI, T2-weighted image. The right orbital cavity, external ocular muscles, and optic nerve are highly enhanced in the T_2_-weighted images and in the T_1_-weighted with Gadolinium enhanced images.

T_1_, T_2_, and Gadolinium enhanced magnetic resonance imaging (MRI) was performed on the whole brain, and fat suppressed imaging for the orbit. Diffuse inflammation was detected in the images of the right orbit but not in the left orbit (Fig. **1**) in both coronary and horizontal sections. The orbital cavity had signs of inflammation, the ocular muscles were swollen although not in the muscle cone. The optic nerve in the retrobulbar region had an edematous ring-shaped image by T_1_-weighted with Gadolinium enhanced MRI. Although the optic disc appeared normal, an optic nerve involvement was suspected, and a tentative diagnose of orbital apex syndrome was made.

After 14 days, betamethazone eyedrops were prescribed for the iritis, and after 22 days, 30 mg predonisolone, was prescribed because his visual acuity had decreased to 0.06. After 40 days, his right optic disc was slightly pale. After 48 days, the stellate ganglion was injected with 6 ml of 1% mepivacaine hydrochloride to alleviate the post-herpetic neuralgia, and 27 weekly injections were given for half year. After 59 days, the adduction of the right eye was improved. But then, a 45 prism diopters (PD) right esotropia was observed at the primary position. He did not complain of double vision possibly due to the poor vision of his right eye.

**Fig. (2):**
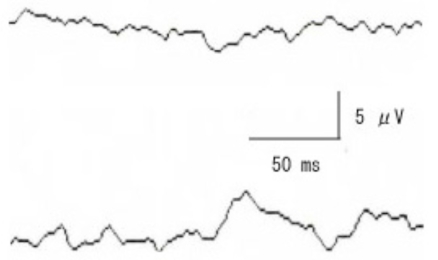
Pattern VECPs elicited by stimulating the right affected eye (upper) and left eye (lower). VECPs are non-recordable when the right affected eye was stimulated while the P100 component is clearly recorded with left eye.

After 108 days, the ptosis had improved, and pattern VECPs were recorded (check size 30 minutes, contrast 90%, reversal rate 3/sec). A response was not elicited by stimulating the right eye, while a P-100 component was clearly obtained by stimulating the left eye (Fig. **2**).

After 115 days, the levator function had improved and the difference in the eyelid opening was 3 mm. After 204 days, the right abduction palsy was slightly improved. After 291 days, the patient had a right ptosis of 4 mm, a slight abduction palsy, and a paralyzed pupil (7 mm). Optic atrophy was still present.

## DISCUSSION

An ophthalmic involvement was found in approximately 50% of the cases of herpes zoster [1] in the Mayo clinic study in 1983 [2] and in the Moorfield Eye Hospital study in 1993 [3]. In both studies, the incidence of keratitis and iritis was >50% of the HZO cases. However, the incidence of an ocular muscle palsy was low. In the Mayo Clinic series, only 3 patients of the 86 patients with HZO had a muscle palsy (3.4%) with the fourth cranial nerve involved in two patients and the sixth cranial nerve in one patient. None of the patients had a third cranial nerve involvement. Of the 1356 patients with HZO in the Moorfield Eye Hospital study, 133 patients (10%) had a muscle palsy with the third cranial nerve involved in 4 of the patients [3]. A total ophthalmoplegia has been reported from a number of clinics [3, 4].

The incidence of optic nerve involvement was even lower than that of ocular muscle palsy with 0.4% in the Moorfield Eye Hospital study [3], and none in the Mayo Clinic study [2]. Several case reports of optic nerve involvement in cases of HZO have been presented from other clinics [6-9]. Most of these had the orbital apex syndrome [5-8] which is also known as optic neuritis/neuropathy associated with complete ophthalmoplegia.

The orbital apex syndrome is caused mainly by neoplastic and granulomatous lesions so that proptosis is usually found. In cases of orbital apex syndrome caused by VZV infections, the incidence of proptosis is lower although Kattah & Kennerdelt [8] reported a 1 mm proptosis in one of 2 patients with HZO.

Recent advances in magnetic resonance imaging (MRI) and pattern VECPs have made them helpful in making a definitive diagnosis of the orbital apex syndrome. However, few reports are available on the MRI findings in cases of orbital apex syndrome caused by VZV infection. Wang *et al.* [6] reported a case of optic neuritis in HZO in which enhanced T_1_-weighted MRI showed that the retrobulbar optic nerve sheath was affected. Quisling *et al.* [10] reported the MRI results in a case of the third cranial nerve palsy caused by herpes zoster. They showed an enhancement of the cisternal and cavernous portion of the third cranial nerve. We have searched Medline for cases reporting the MRI findings in cases of orbital apex syndrome caused by VZV infection, but none was found. Our case is the first report. Our results showed that cases of orbital apex syndrome caused by VZV are characterized by an apparent diffuse inflammatory MR images in the whole orbital cavity, swollen outer ocular muscles just behind the eye but not at the muscle cone, and optic nerve sheath swelling. These MRI findings suggested that the so-called orbital apex syndrome is not caused by a lesion at the muscle cone but is a diffuse neuro-transmitted infection of the virus into the orbit. In addition, the non-recordable pattern VECPs supported a diagnosis of optic neuritis. Our findings verified the pathologic findings of Naumann *et al.* [11] that there was extensive inflammation around the posterior ciliary nerves and vessels.

The clinical course of the orbital apex syndrome caused by VZV was reported to be a complete ophthalmoplegia with recovery in 2 to 18 months [4] after which the optic neuritis progresses to optic atrophy without recovery [12]. Our case showed the same course with a recovery of the ocular motor palsy that progressed from the third cranial nerve to the sixth cranial nerve. The optic neuritis did not improve during the 10 months follow-up period.

Our case shows that ophthalmologists should be aware of the orbital apex syndrome and consider it in cases of optic neuritis caused by VZV infection.
